# Identification of genes associated with testicular germ cell tumor susceptibility through a transcriptome-wide association study

**DOI:** 10.1016/j.ajhg.2025.01.022

**Published:** 2025-02-24

**Authors:** Emilio Ugalde-Morales, Rona Wilf, John Pluta, Alexander Ploner, Mengyao Fan, Mohammad Damra, Katja K. Aben, Lynn Anson-Cartwright, Chu Chen, Victoria K. Cortessis, Siamak Daneshmand, Alberto Ferlin, Marija Gamulin, Jourik A. Gietema, Anna Gonzalez-Niera, Tom Grotmol, Robert J. Hamilton, Mark Harland, Trine B. Haugen, Russ Hauser, Michelle A.T. Hildebrandt, Robert Karlsson, Lambertus A. Kiemeney, Jung Kim, Davor Lessel, Ragnhild A. Lothe, Chey Loveday, Stephen J. Chanock, Katherine A. McGlynn, Coby Meijer, Kevin T. Nead, Jeremie Nsengimana, Maja Popovic, Thorunn Rafnar, Lorenzo Richiardi, Maria S. Rocca, Stephen M. Schwartz, Rolf I. Skotheim, Kari Stefansson, Douglas R. Stewart, Clare Turnbull, David J. Vaughn, Sofia B. Winge, Tongzhang Zheng, Alvaro N. Monteiro, Kristian Almstrup, Peter A. Kanetsky, Katherine L. Nathanson, Fredrik Wiklund, the Testicular Cancer Consortium

**Affiliations:** 1 Department of Medical Epidemiology and Biostatistics, Karolinska Institute, Stockholm, Sweden; 2 Division of Translational Medicine and Human Genetics, Department of Medicine, Perelman School of Medicine, University of Pennsylvania, Philadelphia, PA, USA; 3 Netherlands Comprehensive Cancer Organization, Radboud University Medical Center, Utrecht, the Netherlands; 4 Radboud University Medical Center, Nijmegen, the Netherlands; 5 Department of Surgery (Urology), University of Toronto and The Princess Margaret Cancer Centre, Toronto, ON, Canada; 6 Epidemiology Program, Fred Hutchinson Cancer Center, Seattle, WA, USA; 7 Department of Epidemiology, University of Washington, Seattle, WA, USA; 8 Departments of Preventive Medicine and Obstetrics and Gynecology, Keck School of Medicine at the University of Southern California, Los Angeles, CA, USA; 9 Departments of Urology, Keck School of Medicine at the University of Southern California, Los Angeles, CA, USA; 10 Department of Medicine, University of Padova, Padua, Italy; 11 Department of Oncology, University Hospital Center Zagreb, University of Zagreb School of Medicine, Zagreb, Croatia; 12 Department of Medical Oncology, University Medical Center Groningen, University of Groningen, Groningen, the Netherlands; 13 Human Genotyping Core Unit, Spanish National Cancer Centre (CNIO), Madrid, Spain; 14 Cancer Registry of Norway, Oslo Metropolitan University, Oslo, Norway; 15 Department of Life Sciences and Health, Oslo Metropolitan University, Oslo, Norway; 16 Department of Environmental Health, Harvard T.H. Chan School of Public Health, Boston, MA, USA; 17 Department of Lymphoma and Myeloma, University of Texas, MD Anderson Cancer Center, Houston, TX, USA; 18 Division of Cancer Epidemiology and Genetics, National Cancer Institute, Bethesda, MD, USA; 19 Institute of Human Genetics, University of Regensburg, Regensburg, Germany; 20 Institute of Clinical Human Genetics, University Hospital Regensburg, Regensburg, Germany; 21 Department of Molecular Oncology, Institute for Cancer Research, Oslo University Hospital-Radiumhospitalet, Oslo, Norway; 22 Division of Genetics & Epidemiology, The Institute of Cancer Research, London, UK; 23 William Harvey Research Institute, Queen Mary University, London, UK; 24 Biostatistics Research Group, Population Health Sciences Institute, Faculty of Medical Sciences, Newcastle University, Newcastle, UK; 25 Cancer Epidemiology Unit, Department of Medical Sciences, University of Turin and CPO-Piemonte, Turin, Italy; 26 deCODE Genetics/Amgen, Reykjavik, Iceland; 27 Division of Hematology/Oncology, Department of Medicine, Perelman School of Medicine, University of Pennsylvania, Philadelphia, PA, USA; 28 Department of Growth and Reproduction, Copenhagen University Hospital - Rigshospitalet, Copenhagen, Denmark; 29 Department of Epidemiology, Brown School of Public Health, Brown University, Providence, RI, USA; 30 Department of Cancer Epidemiology, Moffitt Cancer Center and Research Institute, Tampa, FL, USA; 31 Department of Cellular and Molecular Medicine, University of Copenhagen, Copenhagen, Denmark; 32 Abramson Cancer Center, Perelman School of Medicine, University of Pennsylvania, Philadelphia, PA, USA

**Keywords:** testicular germ cell tumors, transcriptome-wide association study, colocalization analysis

## Abstract

Transcriptome-wide association studies (TWASs) have the potential to identify susceptibility genes associated with testicular germ cell tumors (TGCTs). We conducted a comprehensive TGCT TWAS by integrating genome-wide association study (GWAS) summary data with predicted expression models from normal testis, TGCT tissues, and a cross-tissue panel that encompasses shared regulatory features across 22 normal tissues, including the testis. Gene associations were evaluated while accounting for variant-level effects from GWASs, followed by fine-mapping analyses in regions exhibiting multiple TWAS signals, and finally supplemented by colocalization analysis. Expression and protein patterns of identified TWAS genes were further examined in relevant tissues. Our analysis tested 19,805 gene-disease links, revealing 165 TGCT-associated genes with a false discovery rate of less than 0.01. We prioritized 46 candidate genes by considering GWAS-inflated signals, correlations between neighboring genes, and evidence of colocalization. Among these, 23 genes overlap with 22 GWAS loci, with 7 being associations not previously implicated in TGCT risk. Additionally, 23 genes located within 21 loci are at least 1 Mb away from published GWAS index variants. The 46 prioritized genes display expression levels consistent with expected expression levels in human gonadal cell types and precursor tumor cells and significant enrichment in TGCTs. Additionally, immunohistochemistry revealed protein-level accumulation of two candidate genes, *ARID3B* and *GINM1*, in both precursor and tumor cells. These findings enhance our understanding of the genetic predisposition to TGCTs and underscore the importance of further functional investigations into these candidate genes.

## Teaser

The study integrates gene expression prediction models with a testicular germ cell tumor (TGCT) genome-wide association study, identifying 46 risk genes, including novel candidates. The findings enhance the understanding of TGCT genetics, emphasizing the need for further functional studies to inform diagnosis and treatment strategies.

## Introduction

Testicular cancer is the most common type of cancer among males aged 20–40 years in large parts of the world and the second most common type of cancer among males aged 15–19 years.^1^^,^^2^ Testicular germ cell tumors (TGCTs) are the main subtype, with rising global incidence over the last two decades.^3^ TGCTs have a high heritability rate of 40%–50%,^4^^,^^5^^,^^6^^,^^7^ and first-degree male relatives of affected individuals have an 8- to 10-fold higher risk.^8^ TGCTs are thought to arise during fetal testis development, when a subset of primordial germ cells (PGCs) become arrested in an undifferentiated state, maintaining stem cell-like qualities that prevent them from differentiating into spermatogonia.^9^ In this undifferentiated-arrested state, these precursor cells are known as germ cell neoplasia *in situ* (GCNIS). Transformation into TGCTs is thought to begin before puberty and testicular enlargement, when specific genomic alterations—such as the formation of isochromosome 12p—can be identified.^10^^,^^11^ Malignant transformation is then observed in early adulthood, peaking at ages 20–30 years and commonly presenting as a unilateral testicular mass.^12^

Genomic research advancements, particularly through genome-wide association studies (GWASs), have advanced our understanding of the mechanisms underlying many complex traits.^13^ A recent TGCT GWAS meta-analysis^14^ identified 22 additional loci, increasing the total to 78 known TGCT risk loci.^14^^,^^15^^,^^16^^,^^17^^,^^18^^,^^19^^,^^20^ However, the presence of multiple genes within some loci and linkage disequilibrium (LD) complicates the identification of specific causal genes. Transcriptome-wide association studies (TWASs), which utilize expression quantitative trait loci (eQTL) data to link GWAS-identified variants with changes in gene expression, offer a promising method for uncovering potential causal genes.^21^ This approach has been effectively applied in various phenotypes following GWASs.^22^

To identify potential predisposition genes for TGCTs, we performed a TWAS of TGCTs by integrating genetic prediction models of gene expression with GWAS summary statistics. To validate the findings, expression patterns of the identified TWAS genes were explored across relevant tissues.

## Material and methods

### GWAS summary statistics

We utilized GWAS summary statistics from the most recent meta-analysis of TGCT risk, which included 10,156 men with TGCTs and 179,683 male control subjects. All men were of European descent and ascertained from 19 centers.^14^ To ensure a good overlap between GWAS and gene panels, we performed harmonization and imputation of SNP-level *Z* scores using the GWAS summary-imputation pipeline (https://github.com/hakyimlab/summary-gwas-imputation), resulting in 8,638,344 available SNPs and *Z* scores. The analytical set of SNPs consisted of 1,161,660 SNPs that were present in the 1000 Genomes LD reference panel (97.6% overlap). SNP rsIDs were used for matching.

### Gene expression prediction models

We used pre-computed gene expression prediction models from the following source panels: (1) normal testis from the Genotype-Tissue Expression (GTEx)^23^ release v.8, (2) TGCT tissue from The Cancer Genome Atlas (TCGA),^24^^,^^25^ and (3) cross-tissue prediction models derived from 22 GTEx normal tissues (Note S1).^26^ In FUSION,^27^ genetic prediction models were trained using *cis*-SNPs (located within a ±500 kb window from the gene boundary) present in the 1000 Genomes LD reference panel.^28^ Available models were computed using either single SNPs (eQTLs) or a linear combination of multiple *cis*-SNPs (*susie*, *blup*, *elastic-net*, and *lasso* methods). The best model was selected based on the largest out-of-sample *R*^2^ computed via 5-fold cross-validation of each model. In our analysis, only models with both significant *cis*-heritability (*p* < 0.05) and predictive performance (*p* < 0.05 and cross-validation *R*^2^ > 0.01) were considered. The cross-tissue panel consisted of prediction models derived from up to three main cross-tissue features based on sparse canonical correlation analysis (sCCA).^26^ When a gene had more than one significant sCCA feature available, we selected the cross-tissue feature with the highest performance (i.e., cross-validation *R*^2^). In total, 26,710 gene prediction models with significant *cis*-SNP heritability and predictive performance were available: 12,195 from normal testis, 1,254 from TGCTs, and 13,261 from the cross-tissue panel.

### Summary-statistics-based TWAS

The summary-statistics-based TWAS was performed using FUSION software,^27^ which implements a correlation test between genetically predicted gene expression, by using the per-SNP LD-adjusted expression weights learned for the prediction models, and common polygenic predisposition to TGCT, as captured by the per-SNP GWAS *Z* score, across the *cis*-SNPs of a gene of interest. SNP LD was estimated using 489 samples of European ancestry from the 1000 Genomes reference panel.^28^ The statistical significance threshold for the summary-based correlation test was set at a false discovery rate (FDR) of 0.01 over the total number of tests. A TWAS hit was considered as overlapping with any of 78 previously reported TGCT risk loci^14^ if the transcription start site of the gene resided within a ±1 Mb window around the top GWAS SNP.

### TWAS conditional on GWAS effects

To control for inflation of TWAS statistics due to strong SNP-trait signals and extensive LD and to prioritize the observed associations, a conservative gene permutation test was applied on the FDR-significant TWAS hits. The test randomly shuffles expression weights (i.e., sampling with replacement) and recomputes an empirical association statistic conditional on the GWAS effects at the locus.^27^ An effective number of 100,000 permutations were performed on each test, and the significance level was set at 0.05.

### TWAS joint-conditional analysis

We organized genes that passed summary-based and permutation-based correlation tests into separate genetic loci, defined as non-overlapping genomic regions containing neighboring genes within a 100 kb overlap window from their boundaries. Joint-conditional analysis was performed at each locus to identify conditionally independent genes following the FUSION post-process function (http://gusevlab.org/projects/fusion/) (Note S2). The significance threshold for gene selection at each locus was set at a nominal level of 0.05 for the joint-conditional *p* value and Bonferroni adjusted by the number of TWAS significant genes located on the same chromosome.

### Colocalization analysis

Colocalization analysis was performed on the set of conditionally independent TWAS genes in order to identify gene-trait associations supported by shared genetic variants. For this purpose, we employed COLOC, a Bayesian method to estimate the posterior probabilities (PPs) of different variant configurations across two traits.^29^ For each gene, we utilized *cis*-SNP summary statistics for both gene expression (eQTL weights) and TGCT risk (GWAS *Z* scores) to test the hypothesis of a shared causal variant (H4). Effect sizes were estimated based on the assumption that the standard error is inversely proportional to the square root of the sample size. For the TGCT GWAS dataset, we also incorporated the proportion of affected individuals (*n* = 10,156) as reported by Pluta et al.^14^ A significance threshold was set at PP4 > 0.5 to determine colocalization.

### GWAS association conditional on TWAS effect

Following Mancuso et al.,^30^ we evaluated residual association between SNPs at each TWAS risk locus and TGCT after adjusting for the predicted gene expression-TGCT association strength. Initially, the leading genes was derived as detailed in the joint-conditional TWAS analysis. Next, GWAS *Z* scores were imputed for each SNP at the locus conditioned on joint TWAS *Z* scores of selected genes. The resulting residual GWAS *Z* score was standardized to express remaining SNP-TGCT associations after conditioning on the predicted gene expression association.

### Gene expression across human gonadal development, GCNIS, and TGCT

We utilized public expression datasets from human gonadal development, GCNIS, and TGCTs to study the expression patterns of TWAS-identified genes.

### Human gonadal development

Preprocessed and annotated gonadal development gene expression data from human embryos (female: *n* = 33; male: *n* = 22)^31^ were downloaded from The Reproductive Cell Atlas (https://www.reproductivecellatlas.org/gonads.html). We used these data to evaluate whether TWAS candidate genes were found to be expressed in germ cell lineages at different developmental stages (i.e., PGCs, pre-spermatogonia, and pre-oocytes), as well as in male germ cells and somatic cells of the testis (Sertoli and fetal Leydig). In total, the expression levels of 3,764 PGCs, 621 pre-spermatogonia cells, 812 pre-oocyte cells, 3,624 germ cells, 34,927 Sertoli cells, and 2,420 fetal Leydig cells were obtained for 13,963 of the 19,805 genes included in the TWAS analysis. As described by the authors, cells from doublets and those with high mitochondrial content were excluded from the analysis. After filtering, 3,739 PGCs, 486 pre-spermatogonia cells, 757 pre-oocyte cells, 2,883 germ cells, 15,273 Sertoli cells, and 1,769 fetal Leydig cells remained. We defined “no detectable gene expression” as cells with zero expression values. To estimate the average gene expression, values were variance scaled for each cell type, separately.

### Precursor data (GCNIS)

Normalized microarray expression data from six GCNIS samples previously published by Sonne et al.^32^ were obtained from the publicly available ArrayExpress database (https://www.ebi.ac.uk/biostudies/arrayexpress/studies/E-TABM-488). The expression levels were log10 transformed, and the average values were obtained for samples characterized as cultured human embryonic stem cells (ESCs), microdissected spermatogenic tubules, and microdissected tubules containing GCNIS.

### TGCT expression data

RNA sequencing (RNA-seq) data from 150 TGCT samples previously published by TCGA network in Shen et al.^25^ were obtained from the Broad Institute FireBrowse portal (http://firebrowse.org/?cohort=TGCT&download_dialog=true). The preprocessed data were converted to transcripts per million and quantile normalized using the R package PreProcessCore (https://bioconductor.org/packages/release/bioc/html/preprocessCore.html), normalizing each sample to its mean expression across all genes. For each histopathology group (seminomas, *n* = 68; non-seminomas, *n* = 79), the log10-transformed gene expression levels were averaged.

### Enrichment of gene expression

We used a permutation approach to test whether genes identified by TWASs were more likely to be expressed at different stages of TGCT pathogenesis (normal gonadal development, GCNIS, and TGCTs). To this end, we compared the median expression values observed for the set of leading genes (Bonferroni-adjusted joint-conditional *p* < 0.05; see above) to the median expressions for 10,000 random sets of genes of the same size, sampled from the subset of genes included in both the TWAS analysis and the respective expression dataset. For single-cell data, we also compared the median proportion of cells with non-zero expression values.

### Pathway enrichment analysis

We conducted enrichment analysis using PathfindR,^33^ following default settings. PathfindR is an active-subnetwork-oriented approach that leverages annotated protein-protein interaction (PPI) networks to identify clusters of disease-relevant genes that interact within active subnetworks. These subnetworks are then subjected to enrichment analysis via a one-sided hypergeometric test, using the PPI network as the background set. The analysis is performed iteratively (default of 10 iterations), with results reporting the number of times a pathway is enriched and the range of adjusted *p* values across iterations. For this analysis, we utilized Reactome^34^^,^^35^ as the pathway database and BioGRID^36^ for the PPI network. The input data consisted of gene symbols and FDR-adjusted *p* values for the protein-coding TWAS hits with an FDR threshold of <0.01. The significance threshold for pathway enrichment was set at a Bonferroni-adjusted *p* value of <0.05.

### Immunohistochemistry

As a proof of concept, we investigated the presence of two specific TWAS-identified genes at the protein level. We selected one gene from regions not implicated by GWASs, along with another gene from a GWAS region that had not previously been associated with TGCT risk. Following an assessment of commercially available antibodies, ARID3B (from a non-GWAS region) and GINM1 (from a GWAS region) were chosen for analysis. Immunohistochemistry was conducted as previously described.^37^ Tissue sections were obtained from the tissue biobank at the Department of Growth and Reproduction (Rigshospitalet, Copenhagen, Denmark) containing orchiectomy specimens from individuals with testicular cancer (The Danish Data Protection Agency, permit number J.nr. 2001-54-0906). Informed consent was provided by all tissue donors. The use of human samples was approved by the regional medical research ethics committee of the capital region of Copenhagen (H-16019637). Fresh testicular tissue was fixed in our in-house fixative (7.4% formaldehyde, 4% acetic acid, 2% methanol, 0.57% sodium phosphate [dibasic], and 0.11% potassium phosphate [monobasic]) overnight (for at least 16 h) at 4°C, dehydrated, and embedded in paraffin. Immunohistochemistry was conducted on 4 μm sections as previously described.^7^ Briefly, tissue sections were subjected to heat-induced antigen retrieval in a pressure cooker (medical decloaking chamber, Biocare, Concord, CA, USA) in citrate buffer (ARID3B) or in a Tris-EGTA buffer (GINM1, D2-40, and SOX2; 10 mM Tris, 1 mM EDTA, and 0.05% Tween 20 [pH 8.5]) at 110°C for 30 min. Endogenous peroxidase was blocked with 1% (v/v) H_2_O_2_ in methanol for 30 min. Unspecific staining was blocked using 0.5% skimmed milk in Tris-buffered saline (TBS) for 30 min. Sections were incubated overnight at 4°C with primary antibodies against ARID3B (NBP2-33596, Novus Biologicals, San Diego, CO, USA) diluted 1:75, GINM1 (PA5-59671, Thermo Fisher Scientific, Waltham, MA, USA) diluted 1:400, D2-40 (M3619, Dako, Denmark) diluted 1:2,500, SOX2 (AF2018, R&D Systems, Minneapolis, MN, USA) diluted 1:150, or the negative control normal rabbit immunoglobulin (Ig)G (PP501P, Origine, Rockville, MD, USA) diluted 1:75 (for ARID3B) or 1:400 (for GINM1) in a humidified chamber and then incubated for 30 min with the species-specific ImmPRESS HRP (peroxidase) secondary antibodies (Vector Laboratories, Newark, CA, USA). Between all steps (except after the blockage of unspecific staining), the sections were washed in TBS. Visualization was performed using ImmPACT AEC peroxidase (HRP) substrate (Vector Laboratories). The sections were subsequently counterstained with Mayer’s hematoxylin and mounted with Aquatex mounting medium (Merck KGaA, Germany). The slides were scanned using a Hamamatsu NanoZoomer v.2.0 (Hamamatsu Photonics, Japan). Protein accumulation of GNM1 in precursor and tumor cells is illustrated in Figure S2. Negative controls and markers specific to tumor components are shown in Figure S3.

## Results

### TWAS of TGCT

**Figure 1:**
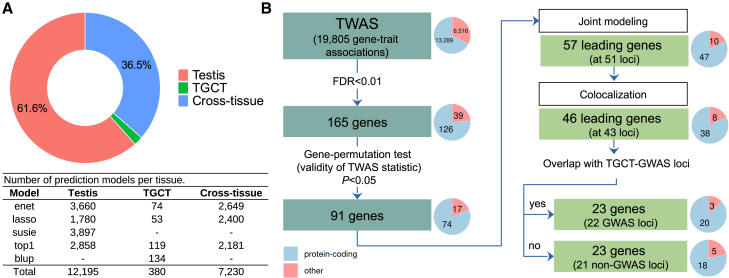
Overview of the study design (A) Prediction models for heritable gene expression included in the analysis. Normal testis from the GTEx panel was selected as the primary target tissue, complemented by additional models from TGCTs from TCGA and cross-tissue features from 22 normal tissues from GTEx (mutually exclusive gene models selected in that order). Prediction models: blup, best linear unbiased predictor; enet, elastic-net regression; lasso, least absolute shrinkage and selection operator regression; susie, sum of single effects regression; top1, single best eQTL. (B) TWAS workflow to identify genes associated with predisposition to TGCTs. Significant genes were defined using a false discovery rate less than 0.01. Robust associations were identified using a gene permutation test *p* < 0.05. Genes were jointly modeled at each locus to identify conditionally independent genes (Bonferroni-adjusted joint-conditional *p* < 0.05). Colocalization analysis was subsequently conducted using a posterior probability (PP4) threshold of >0.5 to identify genes likely influenced by a shared causal variant affecting both gene expression and TGCT risk.

To identify TGCT-associated genes, we conducted TWAS analyses using GTEx and TCGA data against TGCT risk variants (Figure 1A). Our analysis workflow prioritized available prediction models from tissues most biologically related to TGCTs to minimize potential false positive findings and increase statistical power. We selected 12,195 models from normal testis (main target tissue), complemented by 380 models from TGCTs and 7,230 from cross-tissue models capturing common regulatory features of gene expression among 22 normal tissues (testis included). Among these genes, the average *cis*-SNP heritability of expression was 24.5%, and the average predictive performance standardized by SNP heritability was 66.5% (Figure S1).

**Figure 2:**
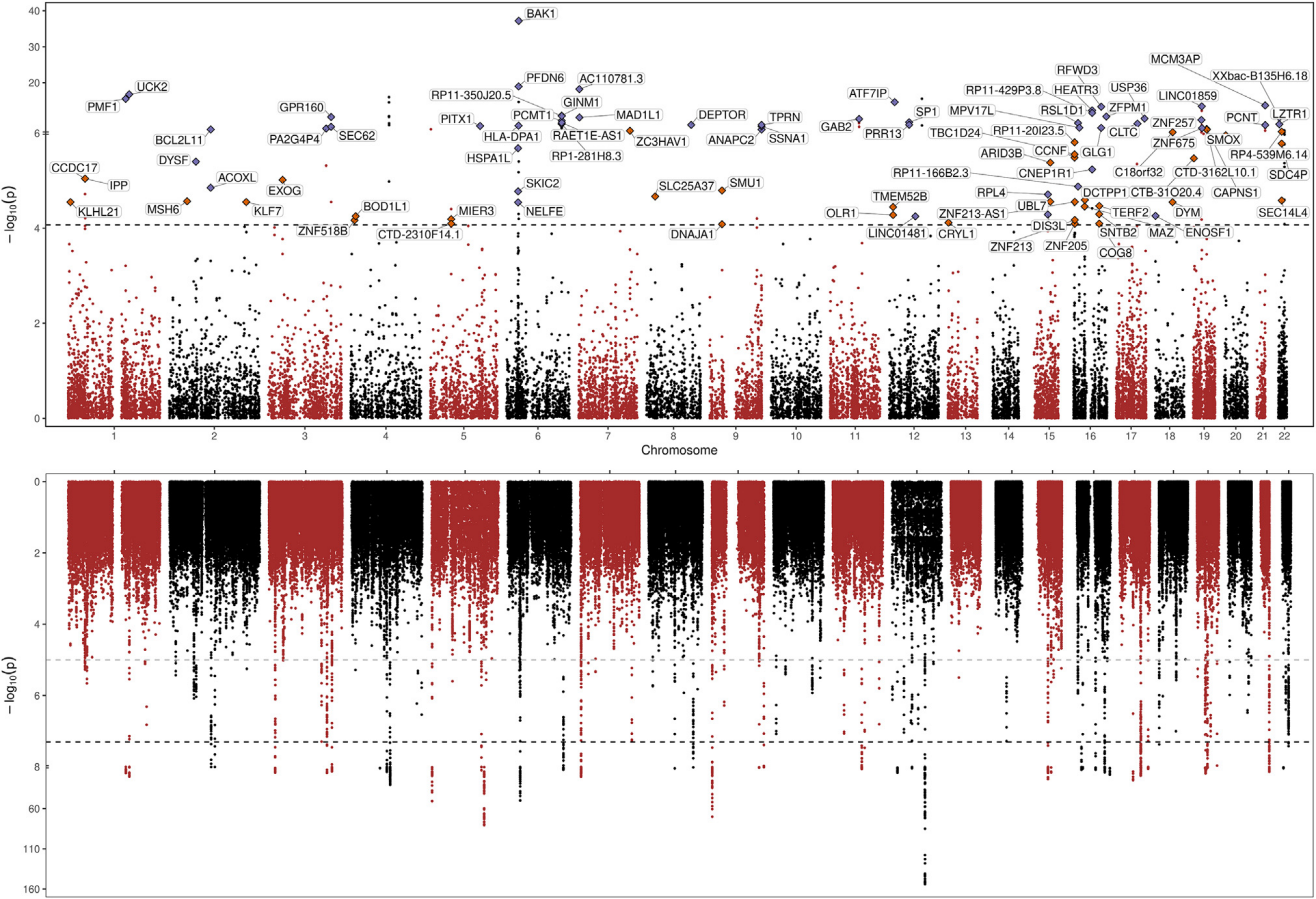
Manhattan/Miami plot summarizing the TWAS gene-TGCT associations in relation to GWAS signals Top: summary of TWAS results showing the strength of association of TGCT genetic risk for 19,805 genes. Named TWAS hits indicated by diamonds were independent of GWAS signals (i.e., permutation *p* < 0.05); color indicates whether the TWAS hit is located at a GWAS locus (purple diamond) or at a non-GWAS region (red diamond). The dotted horizontal line represents the significance threshold at false discovery rate < 0.01. Bottom: strength of SNP-level associations for TGCT risk (−log10 *p* value) as reported in the TGCT GWAS meta-analysis results.^14^ Shown are 1,161,660 SNPs that were included in the FUSION prediction models (i.e., SNPs that are present in the 1000 Genomes reference panel). Dotted horizontal lines indicate the GWAS-suggestive threshold (*p* < 1 × 10^−5^) in light gray and the GWAS-significance threshold (*p* < 5 × 10^−8^) in dark gray.

We tested 19,805 gene-disease associations for TGCT risk (Table S1) and visualized the results in Figure 2. Of these, 165 candidate genes were associated at FDR < 0.01 (Table S2). The majority (87, 52.7%) were from predicted expression in normal testis, 68 (41.2%) from the cross-tissue panel, and 10 (6.1%) from TGCT tissue. Pathway enrichment analysis, performed on the 123 protein-coding genes identified from the 165 TWAS hits that mapped to established protein interaction networks (as detailed in the material and methods), identified significant over-representation in 154 Reactome pathways (Table S3). Notably, pathways involved in cell cycle regulation, particularly those related to chromosomal segregation, were prominently enriched. Specifically, the mitotic spindle checkpoint and separation of sister chromatids pathways showed substantial fold enrichments of 9.82 and 6.35, respectively, with strong statistical significance (*p* = 8.80 × 10^−7^ and 5.1 × 10^−6^). In addition to cell-cycle-related pathways, the analysis underscored the role of DNA repair mechanisms, evidenced by the enrichment of the SUMOylation of DNA replication proteins pathway (fold enrichment of 8.04, *p* = 4.7 × 10^−5^).

To mitigate potential TWAS statistic inflation from GWAS signals, we performed gene-wise permutation tests, identifying 91 out of 165 TWAS candidate genes as robustly associated with TGCTs (Table S2; Figure 1B). These genes coalesce into 51 genomic loci, with 25 loci harboring a single gene and 26 accommodating multiple genes. Subsequently, joint-conditional TWASs within loci with multiple genes identified 57 leading genes showing robust and independent associations with TGCTs (Tables 1, S4, and S5; Figure 1B). Following this analysis, only five loci contained multiple genes. Colocalization analysis was conducted on the 57 genes remaining after joint-conditional analysis. Using a PP (PP4) threshold greater than 0.5, evidence was found that 46 genes harbor a shared causal variant influencing both gene expression and TGCT risk. Among these 46 leading genes, the mean PP4 was 0.87 (interquartile range: 0.82–0.98). Of these, 23 genes were situated within 22 previously identified TGCT GWAS risk loci, while the other 23 genes were mapped to 21 regions not previously associated with TGCT risk in GWASs (Table 1).

**Table 1:** TWAS joint conditionally independent genes associated with TGC

**Cytoband**	**Gene symbol**	**Position (hg38)**	**Gene type**^a^	**Tissue**	**Joint *Z* score**	**Joint** ***p* value**	**PP4**	**GWAS locus**^b^
1p36.31	*KLHL21*	chr1:6,590,723–6,614,607	PC	TGCT	4.19	2.83 × 10^−5^	0.608	N
1p34.1	*CCDC17*	chr1:45,620,043–45,624,057	PC	cross-tissue	−4.44	9.08 × 10^−6^	0.905	N
1q22	*PMF1*	chr1:156,212,992–156,240,042	PC	TGCT	−8.17	3.02 × 10^−16^	0.927	Y^c^
1q24.1	*UCK2*	chr1:165,827,755–165,908,109	PC	testis	8.52	1.62 × 10^−17^	0.998	Y^c^
2p16.3	*MSH6*	chr2:47,695,529–47,810,101	PC	testis	−4.20	2.67 × 10^−5^	0.916	N
2p13.2	*DYSF*	chr2:71,453,721–71,686,768	PC	testis	−4.61	4.03 × 10^−6^	0.948	Y
2q13	*BCL2L11*	chr2:111,119,377–111,168,447	PC	cross-tissue	5.33	9.95 × 10^−8^	0.990	Y^c^
2q33.3	*KLF7*	chr2:207,074,136–207,167,267	PC	testis	4.19	2.79 × 10^−5^	0.933	N
3p22.2	*EXOG*	chr3:38,496,126–38,525,951	PC	testis	−4.43	9.64 × 10^−6^	0.921	N
3q25.31	*PA2G4P4*	chr3:156,809,550–156,810,732	Pps	cross-tissue	5.44	5.32 × 10^−8^	0.545	Y
3q26.2	*GPR160*	chr3:170,037,928–170,085,403	PC	testis	6.63	3.31 × 10^−11^	0.863	Y^c^
4p16.1	*ZNF518B*	chr4:10,439,873–10,457,408	PC	cross-tissue	−3.98	6.81 × 10^−5^	0.932	N
4p15.33	*BOD1L1*	chr4:13,568,737–13,627,723	PC	cross-tissue	4.03	5.60 × 10^−5^	0.654	N
5q11.2	*MIER3*	chr5:56,919,601–56,971,675	PC	testis	3.99	6.47 × 10^−5^	0.540	N
6p21.33	*HSPA1L*	chr6:31,809,618–31,815,065	PC	cross-tissue	4.75	2.08 × 10^−6^	0.850	Y
6p21.31	*BAK1*	chr6:33,572,546–33,580,293	PC	TGCT	−9.02	1.91 × 10^−19^	0.683	Y^c^
6q25.1	*GINM1*	chr6:149,566,293–149,591,748	PC	testis	6.76	1.40 × 10^−11^	0.863	Y
7p22.3	*AC110781.3*	chr7:1,838,585–1,849,931	Ant	testis	−8.89	5.93 × 10^−19^	0.977	Y
7q34	*ZC3HAV1*	chr7:139,043,519–139,109,719	PC	cross-tissue	5.17	2.29 × 10^−7^	0.979	N
8q24.12	*DEPTOR*	chr8:119,928,399–120,050,913	PC	TGCT	−5.84	5.21 × 10^−9^	0.913	Y^c^
9p21.1	*SMU1*	chr9:33,041,763–33,076,659	PC	testis	4.31	1.61 × 10^−5^	0.874	N
9q34.3	*TPRN*	chr9:137,191,616–137,200,798	PC	testis	−5.84	5.34 × 10^−9^	0.994	Y^c^
11q14.1	*GAB2*	chr11:78,215,296–78,418,348	PC	cross-tissue	6.44	1.18 × 10^−10^	0.994	Y^c^
12p13.2	*TMEM52B*	chr12:10,170,541–10,191,801	PC	testis	−4.13	3.59 × 10^−5^	0.701	N
12p13.1	*ATF7IP*	chr12:14,365,631–14,502,935	PC	cross-tissue	−7.92	2.43 × 10^−15^	0.949	Y^c^
12q13.13	*SP1*	chr12:53,380,175–53,416,446	PC	testis	6.12	9.58 × 10^−10^	0.952	Y^c^
13q12.11	*CRYL1*	chr13:20,403,666–20,525,857	PC	testis	−3.96	7.64 × 10^−5^	0.543	N
15q24.1	*ARID3B*	chr15:74,541,176–74,598,131	PC	testis	−4.60	4.18 × 10^−6^	0.973	N
16p13.3	*RP11-20I23.5*	chr16:2,575,627–2,577,373	Tec	cross-tissue	4.48	7.48 × 10^−6^	0.956	N
16p13.3	*ZNF213-AS1*	chr16:3,110,459–3,134,882	Ant	testis	3.91	9.36 × 10^−5^	0.853	N
16p13.11	*MPV17L*	chr16:15,395,753–15,413,268	PC	cross-tissue	5.53	3.12 × 10^−8^	0.964	Y^c^
16p11.2	*DCTPP1*	chr16:30,423,618–30,424,533	PC	cross-tissue	−4.21	2.55 × 10^−5^	0.870	N
16q12.1	*HEATR3*	chr16:50,065,940–50,106,387	PC	testis	7.23	4.80 × 10^−13^	0.996	Y^c^
16q22.1	*TERF2*	chr16:69,355,560–69,408,571	PC	cross-tissue	−4.14	3.41 × 10^−5^	0.782	N
16q24.2	*ZFPM1*	chr16:88,453,316–88,537,016	PC	testis	6.68	2.47 × 10^−11^	0.812	Y^c^
17q25.3	*USP36*	chr17:78,787,380–78,841,441	PC	testis	6.48	9.12 × 10^−11^	0.914	Y^c^
18q21.1	*C18orf32*	chr18:49,477,249–49,487,252	PC	testis	3.79	1.48 × 10^−4^	0.545	N
19p13.3	*CTB-31O20.4*	chr19:1,822,088–1,824,542	Ptr	testis	4.65	3.40 × 10^−6^	0.786	N
19p12	*ZNF257*	chr19:22,052,451–22,091,480	PC	testis	5.92	3.28 × 10^−9^	0.980	Y^c^
19p12	*ZNF675*	chr19:23,525,630–23,687,220	PC	cross-tissue	−5.11	3.18 × 10^−7^	0.983	Y
19q13.12	*CAPNS1*	chr19:36,139,574–36,150,353	PC	testis	−3.05	2.28 × 10^−3^	0.992	N
19q13.12	*CTD-3162L10.1*	chr19:36,304,579–36,312,668	Lnc	testis	−2.93	3.39 × 10^−3^	0.993	N
20p13	*SMOX*	chr20:4,120,979–4,187,747	PC	TGCT	−4.87	1.11 × 10^−6^	0.551	N
21q22.3	*MCM3AP*	chr21:46,236,828–46,286,297	PC	testis	7.65	1.97 × 10^−14^	0.984	Y^c^
22q11.21	*XXbac-B135H6.18*	chr22:20,981,360–20,981,755	Lnc	testis	−6.59	4.47 × 10^−11^	0.999	Y
22q12.2	*RP4-539M6.14*	chr22:30,475,363–30,492,804	Ant	testis	5.07	4.03 × 10^−7^	0.989	N

Joint-conditional results for 46 out of 57 independent genes surpassing colocalization analysis at the posterior probability of a common genetic variant (PP4 > 0.5), located at 43 genomic regions. Joint analysis was based on 91 genes with robust association (FDR_TWAS_ < 0.01 and gene permutation test *p* < 0.05) mapped onto 51 non-overlapping regions. PC, protein-coding; pps, processed pseudogene; ant, antisense; lnc, long intergenic non-coding RNA; tec, to be experimentally confirmed; ptr, processed transcript; Y, yes; N, no.

a ENSEMBL biotype annotation.

b Overlap with a GWAS loci.

c Gene previously reported as implicated in TGCT risk.

### GWAS association conditional on TWAS effect

**Figure 3:**
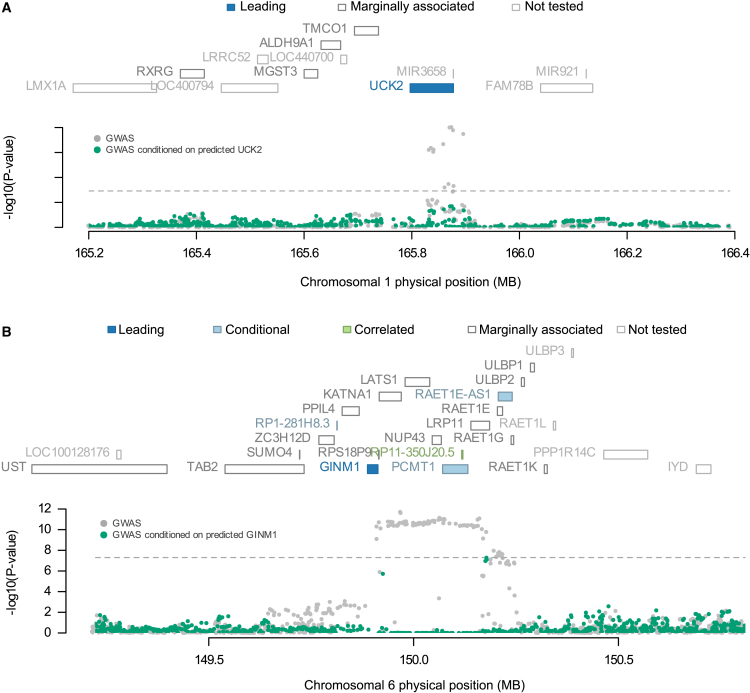
GWAS signals explained by top leading TWAS genes: *UCK2* and *GINM1* *UCK2* (A) and *GINM1* (B) have the lowest permutation *p* values among the 46 leading genes in Table 1. Each image consists of a top and bottom image. The top image shows all genes located at the risk locus according to the genomic position (hg19). Based on the joint modeling, genes are labeled as leading in dark blue (joint-conditionally independent), conditional on leading genes(s) in light blue, or correlated with leading genes in green (predicted expression *R*^2^ > 0.9); marginally associated genes (*p*_TWAS_ ≥ 0.01 or *p*_permutation_ ≥ 0.05) and genes not tested (e.g., due to lack of a significant prediction model) are indicated with empty boxes with dark and light outlines, respectively. The bottom image shows results from the SNP-level GWAS-conditional analysis. Each point illustrates the association between a single SNP at the locus and TGCT status: gray points indicate the marginal association of a SNP with TGCT status (GWAS association) and green points indicate the association of the same SNP with TGCTs after conditioning of predicted expression of the leading gene at each locus (here, *UCK2* and *GINM1*). The horizontal dashed line indicates the genome-wide significance threshold (*p* = 5 × 10^−8^).

To assess the proportion of the GWAS signal accounted for by the identified TWAS genes, we investigated the residual SNP association with TGCT after adjusting for the local TWAS signal at each of the 22 GWAS loci (Table S7). The median proportion of GWAS signals explained across the 22 GWAS loci was 86%, with an interquartile range of 75%–92%. In Figure 3, the effect of conditioning on TWAS effects on GWAS signals is illustrated for the two loci containing *UCK2* and *GINM1*, capturing 82% and 100% of the GWAS signal, respectively.

### TWAS genes are expressed at various stages of TGCT development

**Figure 4:**
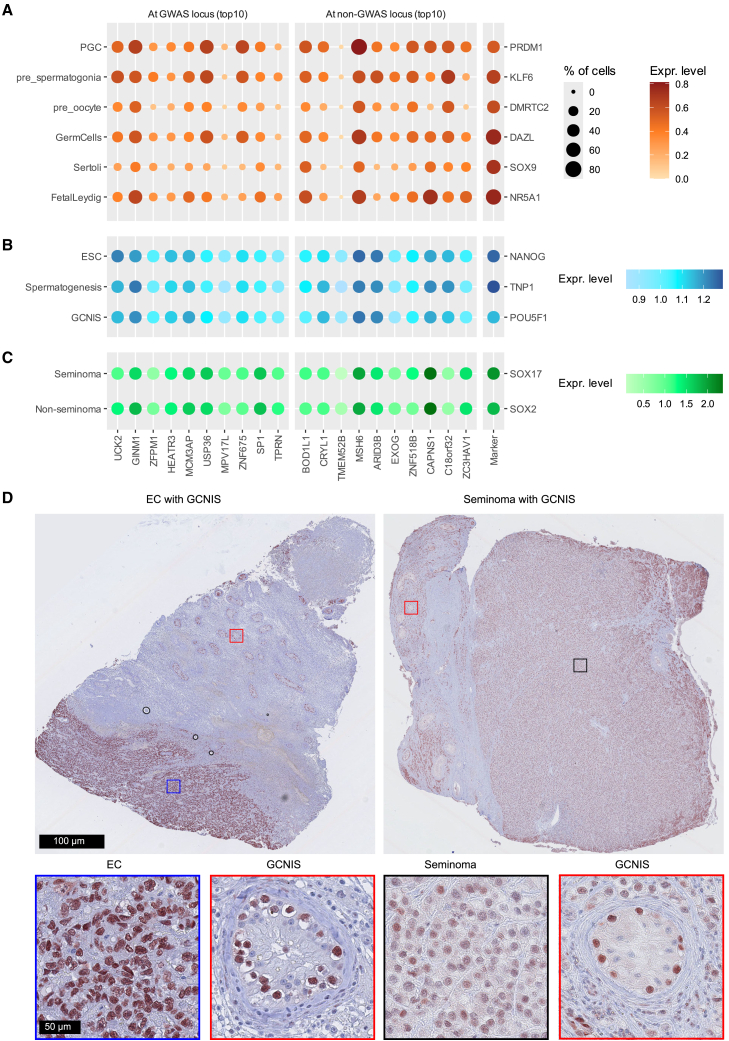
Expression patterns of top leading genes at various tissues (A–C) Expression levels of ten most significant genes among genes overlapping a GWAS locus (left) or not (right). Genes are ordered by strength of evidence (*p*_permutation_) with decreasing significance from left to right; expression levels of tissue-specific marker genes are shown for reference on the right. (A) Human embryonal gonads. Single-cell average gene expression (color panel) and proportion of cells with non-zero expression (dot size) at different stages of germ cell differentiation (primordial germ cell [PGC], pre-spermatogonia, and pre-oocyte cell types) as well as in male germ cells and supporting cells (Sertoli and fetal Leydig). (B) Pre-malignant tissue. Microarray expression from microdissected samples characterized as embryonal stem cells (ESCs), spermatogenesis, and germ cell neoplasia *in situ* (GCNIS). The color panel indicates the log10-average expression levels at each condition. (C) TGCTs. The color panel indicates the log10-average expression levels. (D) Protein localization of ARID3B, in GCNIS adjacent to a non-seminoma (embryonal carcinoma [EC]), and GCNIS adjacent to a seminoma. For each TGCT subtype, both the tumor and GCNIS component are shown in greater magnification at the bottom. Scale bars indicate 100 and 50 μm, respectively.

We examined expression patterns of the 46 conditional independent TWAS genes showing evidence of colocalization compared to non-TWAS genes in datasets covering human gonadal development, GCNIS, and TGCT samples using a permutation method (see material and methods; Table S8). The expression levels and proportions of cells expressing the 46 leading TWAS genes were consistent with the anticipated expression levels and cell proportions within specific human gonadal cell types, including germ cell differentiation, germ cells, and supporting cells. Similarly, comparable expression levels were detected in microdissected GCNIS samples. Furthermore, a significant enrichment was observed in both seminoma (*p* = 0.004) and non-seminoma (*p* = 0.01) tumor tissue samples. Expression patterns of the ten most significant TWAS genes within GWAS and non-GWAS loci related to specific cell type marker genes are illustrated in Figures 4A–4C.

### Protein-level abundance

Immunohistochemistry was performed to investigate protein-level accumulation of two candidate genes, *ARID3B* (Figure 4D) and *GINM1* (Figure S2), in relevant tissues. The proteins encoded by these two genes exhibited elevated accumulation in precursor (GCNIS) cells adjacent to both seminoma and non-seminoma, as well as within seminoma. ARID3B protein levels also were detected in non-seminoma (embryonal carcinoma), whereas GINM1 protein levels were not detected. Negative controls and markers specific to tumor components are shown in Figure S3.

## Discussion

We conducted a TGCT-focused TWAS using GWAS summary data and genetically predicted expression models across multiple tissues. Over 19,000 prediction models were explored, identifying 165 TGCT-associated genes with FDR < 0.01. Following a conservative permutation test, joint-conditional analyses, and colocalization assessment, 46 leading genes remained associated with TGCTs. Among them, 23 genes were in known GWAS loci, 7 of which have not been previously implicated in the risk of TGCTs, and 23 genes were in 21 loci not previously identified by GWASs.

Gene expression prediction models were trained in normal and tumor testicular tissue and a set of 22 normal tissues. By employing this multi-tissue methodology, we identified an increased number of genes compared to using testis alone. For example, the multi-tissue approach identified *ATF7IP* as implicated in TGCT etiology.^38^ ATF7IP acts as a transcription factor, facilitating the expression of *TERT*, also implicated as being involved in TGCT susceptibility through GWASs, and its corresponding RNA component, *TERC*.^39^ ATF7IP also interacts with SP1,^39^ which was also identified in this study, linking the pathway of chromatin organization to TGCT susceptibility.

Our study identified 23 genes across 21 loci previously unreported in GWASs. Notably, *MSH6* and *ARID3B*, identified through TWASs, are critical in DNA damage repair pathways linked to TGCT pathogenesis. *MSH6*, a key DNA mismatch repair gene,^40^ is highly expressed in PGCs,^31^^,^^41^ and its mutations are linked to Lynch syndrome.^40^^,^^41^^,^^42^ It also contributes to genomic imprinting,^43^ a critical process in germ cell development connected to TGCTs by promoting cancer stemness.^44^^,^^45^^,^^46^ ARID3B, known for its role in chromatin remodeling and gene expression regulation,^47^ promotes cancer stemness and tumor growth in ovarian cancer xenografts.^48^^,^^49^ It regulates stem cell markers such as *POU5F1* (*OCT4*) and *SOX2*,^50^^,^^51^ associated with GCNIS and embryonal carcinoma.^32^^,^^44^^,^^45^ Notably, in mouse embryonic development, *Arid3b* is highly expressed specifically in developing testes, suggesting a similar role for its highly conserved human counterpart.^52^

**Figure 5:**
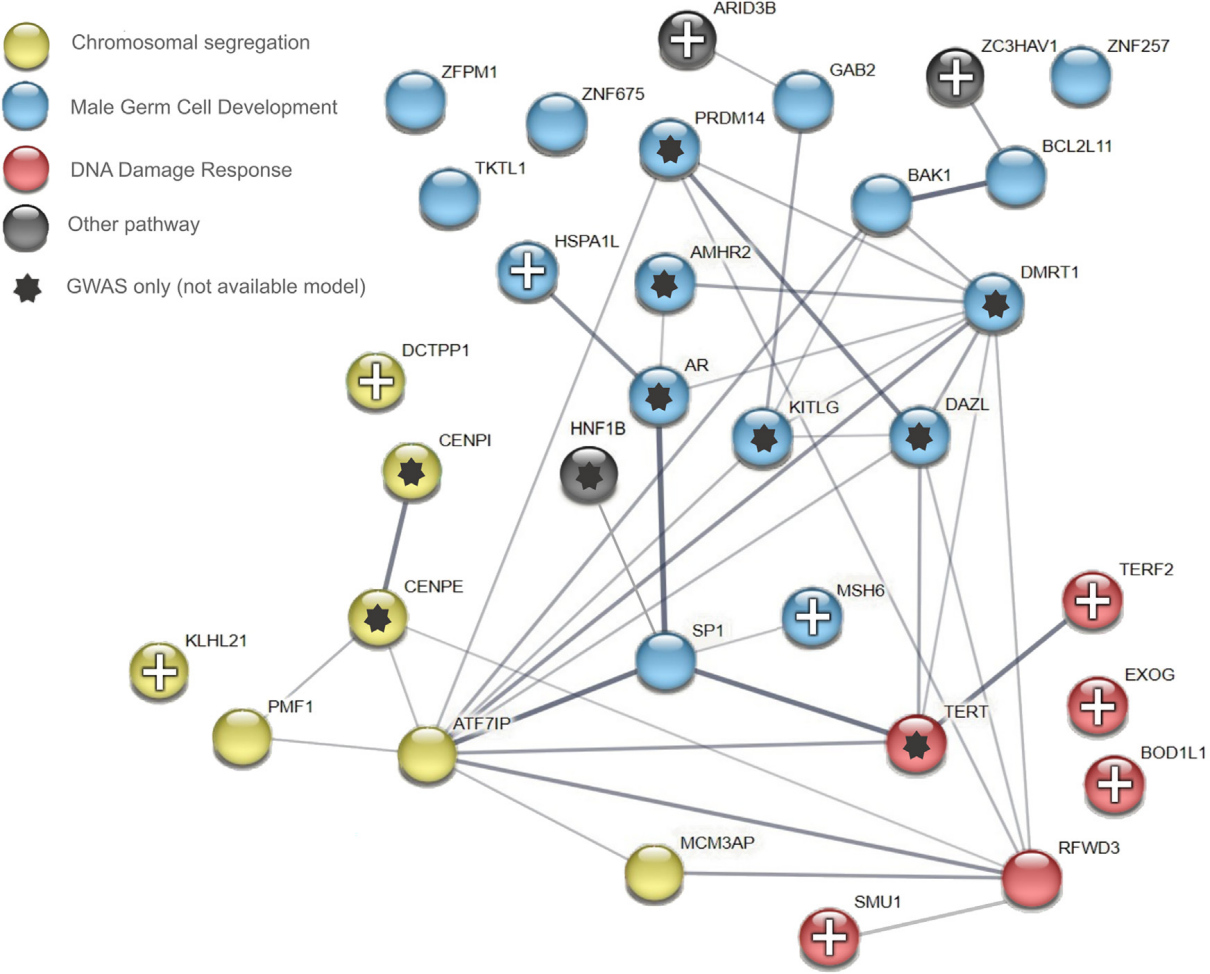
Network interactions and TGCT-relevant annotations for TWAS leading genes A protein-protein interaction network in STRING (string-db.org) for TWAS leading genes along with genes implicated by GWASs^14^ that were not included in the current study due to lack of a prediction model. Disconnected gene nodes without relevant annotations were removed. Line weights indicate the degree of confidence of an interaction between any two proteins based on STRING presets. Pathway annotation was based on manual curation as described in the discussion and Table S9.

Our pathway enrichment analysis provided additional evidence supporting the involvement of genes associated with chromosomal segregation and the DNA damage response in TGCTs. After carefully reviewing the literature, we annotated TWAS- and GWAS-identified genes in the context of three main pathways relevant to TGCT etiology—male germ cell development, chromosomal segregation, and the DNA damage response (Figure 5; Table S9). Figure 5 includes genes identified through GWASs that could not be evaluated in TWASs because a prediction model was not available. Integration of the findings from both GWASs and TWASs broadens the biological inference and expands the genes in these pathways, in which variation is associated with susceptibility to TGCTs.

We assessed the expression patterns of the identified TWAS genes across human gonadal development, GCNIS, and TGCT samples. Overall, the expression levels of these TWAS genes aligned with the anticipated expression levels in gonadal development and GCNIS samples. Additionally, significant enrichment was observed in both seminoma and non-seminoma tumor tissue samples, supporting a potential causal association between the identified genes and TGCTs. Additionally, through immunohistochemistry, we demonstrated the protein-level accumulation of two candidate genes, *ARID3B* and *GINM1*, in both precursor and tumor cells. *ARID3B* is frequently co-expressed with *MYCN* in germ cell tumors, ESCs, and testis—cell types associated with pluripotency.^53^ This co-expression is known to repress apoptosis and promote cell cycle progression, both critical in cancer development.^54^ The N-glycosylation pattern of GINM1, linked to disease progression in bladder cancer,^55^ has not been previously associated with TGCTs to our knowledge.

Our study has several limitations. Firstly, our expression models, trained on adult tissues, might not capture genes specifically expressed during gonadal development. Secondly, even with a multi-tissue approach, we faced challenges in developing prediction models for some key genes associated with TGCT, such as *KITLG*, which plays a critical role in the disease’s pathogenesis.^56^ Thirdly, the absence of rare variants during model construction may have led to overlooking important TGCT genes identified in sequencing studies.^57^ Additionally, we limited our analysis to autosomal chromosomes, ignoring potentially critical sex-linked genes like *AR*, hypothesized to contribute to TGCT pathogenesis.^14^ Lastly, our study solely focused on overall gene expression, neglecting the influence of RNA splicing on disease etiology, which has been shown to represent a critical link between genetic variation and complex diseases.^58^

### Conclusions

In summary, utilizing TWAS methodology, we have successfully identified numerous genes associated with TGCT susceptibility, warranting further investigation into their functional roles. Our approach has revealed previously unreported susceptibility regions and identified candidate genes within several well-established susceptibility loci. Future investigations employing enhanced TWAS methodologies, encompassing rare coding variants and gene expression levels specific to splicing events, hold the potential to uncover additional genes associated with susceptibility to TGCTs.

## Data and code availability

The code used in this analysis is available at https://github.com/emiuga/TGCT_TWAS. Fusion software, prediction models, and reference LD are available at http://gusevlab.org/projects/fusion/. TGCT GWAS summary data used in this study are available at https://www.ncbi.nlm.nih.gov/projects/gap/cgi-bin/study.cgi?study_id=phs001349.v2.p1.

## Acknowledgments

Please see the supplemental information for acknowledgments.

## Author contributions

F.W., K.L.N., and P.A.K. conceived and supervised the study. E.U.-M. and R.W. performed primary data analysis. E.U.-M., R.W., A.P., R.K., S.B.W., J.P., A.N.M., K.A., P.A.K., K.L.N., and F.W. contributed to the analysis and interpretation of data. M.F., M.D., K.K.A., L.A.-C., C.C., V.K.C., A.F., M.G., J.A.G., A.G.-N., T.G., R.J.H., M.H., T.B.H., R.H., M.A.T.H., L.A.K., J.K., D.L., R.A.L., C.L., S.J.C., K.A.M., C.M., K.T.N., J.N., M.P., T.R., L.R., M.S.R., S.M.S., R.I.S., K.S., D.R.S., C.T., D.J.V., T.Z., K.A., P.A.K., K.L.N., and F.W. contributed phenotypic data and genotypic data from men with and without TGCT. E.U.-M., R.W., F.W., and K.L.N. wrote the manuscript with critical input from all authors. All authors approved the final version of the manuscript.

## Declaration of interests

The authors declare no competing interests.

## Web resources

EMBL’s European Bioinformatics Institute, https://www.ebi.ac.uk/biostudies/arrayexpress/studies/E-TABM-488
FireBrowse, http://firebrowse.org/?cohort=TGCT&download_dialog=true
GitHub, https://github.com/hakyimlab/summary-gwas-imputation
Gusev Lab, http://gusevlab.org/projects/fusion/
NIH, https://www.ncbi.nlm.nih.gov/projects/gap/cgi-bin/study.cgi?study_id=phs001349.v2.p1
The Reproductive Cell Atlas, https://www.reproductivecellatlas.org/gonads.html

## Supplemental information

Document S1. Figures S1–S4 and Notes S1 and S2

Data S1. Tables S1–S10

Document S2. Article plus supplemental information
