# Supplementary material for: Identification of genes associated with testicular germ cell tumor susceptibility through a transcriptome-wide association study
Source: Am J Hum Genet. 2025 Feb 24;112(3):630–43. doi: 10.1016/j.ajhg.2025.01.022 (PMC11947167; doi:10.1016/j.ajhg.2025.01.022)
Supplement: Document S1. Figures S1–S4 and Notes S1 and S2 [file mmc1.pdf]

The American Journal of Human Genetics, Volume *112*

Supplemental information

Identification of genes associated with testicular germ cell tumor susceptibility through a transcriptome-wide association study

Emilio Ugalde-Morales, Rona Wilf, John Pluta, Alexander Ploner, Mengyao Fan, Mohammad Damra, Katja K. Aben, Lynn Anson-Cartwright, Chu Chen, Victoria K. Cortessis, Siamak Daneshmand, Alberto Ferlin, Marija Gamulin, Jourik A. Gietema, Anna Gonzalez-Niera, Tom Grotmol, Robert J. Hamilton, Mark Harland, Trine B. Haugen, Russ Hauser, Michelle A.T. Hildebrandt, Robert Karlsson, Lambertus A. Kiemeney, Jung Kim, Davor Lessel, Ragnhild A. Lothe, Chey Loveday, Stephen J. Chanock, Katherine A. McGlynn, Coby Meijer, Kevin T. Nead, Jeremie Nsengimana, Maja Popovic, Thorunn Rafnar, Lorenzo Richiardi, Maria S. Rocca, Stephen M. Schwartz, Rolf I. Skotheim, Kari Stefansson, Douglas R. Stewart, Clare Turnbull, David J. Vaughn, Sofia B. Winge, Tongzhang Zheng, Alvaro N. Monteiro, Kristian Almstrup, Peter A. Kanetsky, Katherine L. Nathanson, Fredrik Wiklund, and the Testicular Cancer Consortium

SUPPLEMENTAL INFORMATION

Note S1. List of GTEx tissues included in the Cross-tissue prediction models2

Note S2. TWAS joint-conditional analysis3

Figure S1. Genetic prediction models for gene expression in different tissues4

Figure S2. Quantile-quantile P-value plot of the 19,805 TGCT-TWAS association tests5

Figure S3. GINM1 protein expression6

Figure S4. Immunohistochemical markers of tumor components and negative controls7

Acknowledgments8

Note S1. List of GTEx tissues included in the Cross-tissue prediction models [Feng H, et al., Plos Genetics (2021); PMID: **33831007**].

1. Adipose Subcutaneous
2. Adipose Visceral Omentum
3. Artery Aorta
4. Artery Tibial
5. Breast Mammary Tissue
6. Colon Sigmoid
7. Colon Transverse
8. Esophagus Gastroesophageal Junction
9. Esophagus Mucosa
10. Esophagus Muscularis
11. Heart Atrial Appendage
12. Heart Left Ventricle
13. Lung
14. Muscle Skeletal
15. Nerve Tibial
16. Pancreas
17. Skin Not Sun Exposed Suprapubic
18. Skin Sun Exposed Lower leg
19. Stomach
20. Testis
21. Thyroid
22. Whole Blood

Note S2. TWAS joint-conditional analysis

Joint-conditional analyses were carried out to identify independent leading genes at locus levels following the FUSION post-process function (http://gusevlab.org/projects/fusion/). In brief, the analysis consists of two steps: 1) selection of conditionally independent genes and 2) calculation of joint z scores for the independent genes and calculation of conditional z scores on the non-independent genes. The feature selection is a stepwise-forward procedure that starts by selecting the gene with the largest summary-based TWAS statistic at a given risk locus as the leading gene and iteratively updates the TWAS test scores of all remaining genes (i.e. computes conditional TWAS z scores) by subtracting the effects of hitherto selected leading genes; at each step, the unselected gene with the largest updated TWAS statistic is selected, until no unselected gene with a significant residual TWAS association remains, and the selection step terminates. To avoid instability of the selection procedure due to excess collinearity between genes or miss-specified covariance structure, genes in strong correlation with any of the leading genes (predicted expression R2 > 0.9, estimated from the 1000 Genomes LD reference panel) are excluded, as well as genes where an update significantly increased the TWAS statistic.

In the FUSION software, the algorithm iterates over all input genes in the same chromosome. Here, the analysis was performed to compute TWAS joint-conditional estimates for the selected leading genes at each locus (joint z score and p value). For the remaining genes at each locus (i.e., conditionally not significant or excluded), we computed conditional z scores and p values by subtracting the effects of the leading genes. Following the FUSION algorithm, subsets of genes were weighted by their predicted expression covariance structure to control for gene-gene correlations, analogous to LD for SNPs, in all calculations.

The modified version of the FUSION post-process scripts used to perform locus-level analysis and to customize output (e.g. summary tables and plots), are made available through a GitHub repository (https://github.com/emiuga/TGCT_TWAS).

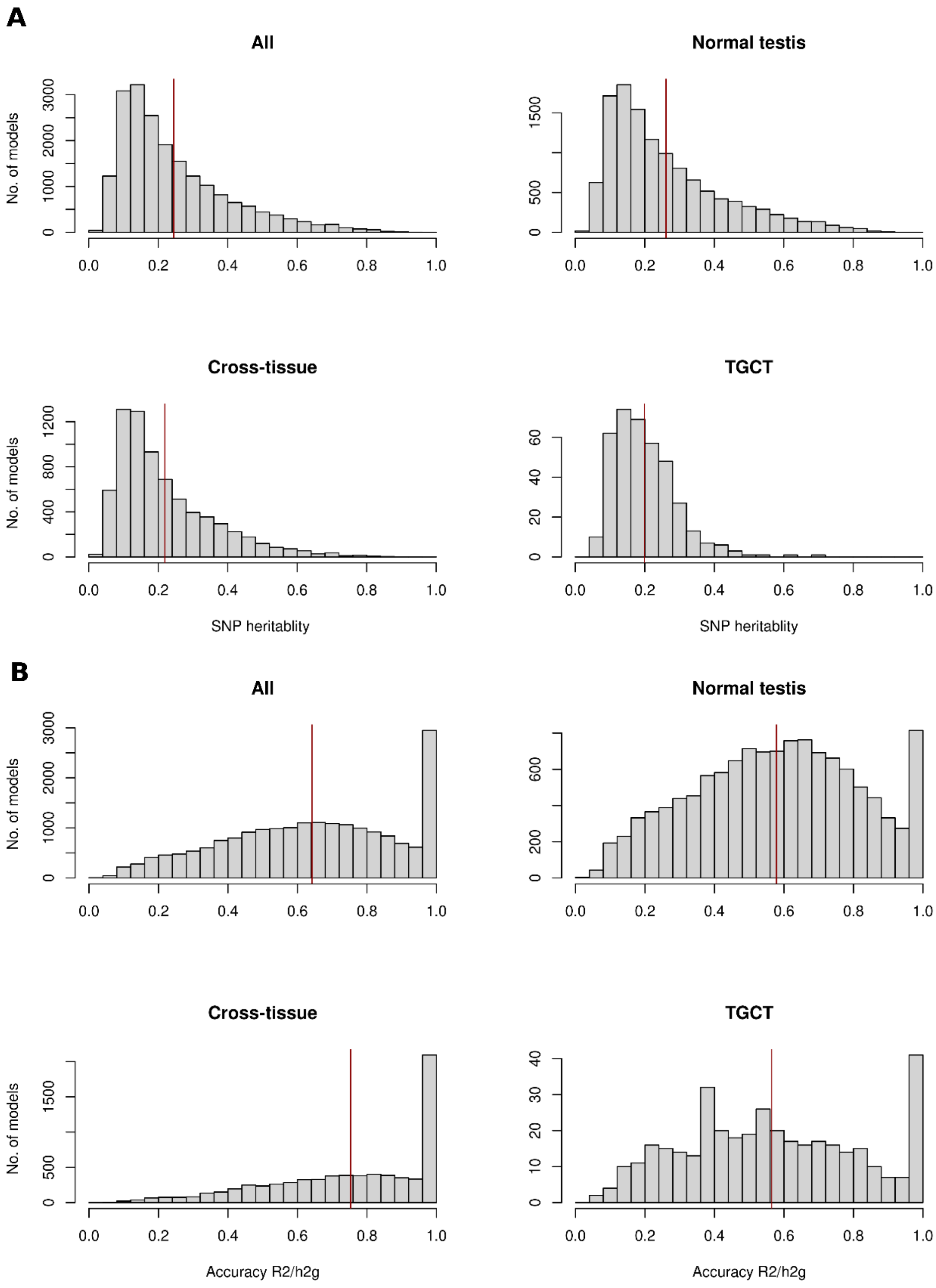

Figure S1. Genetic prediction models for gene expression in different tissues. **a)** SNP-based heritability estimated by cis-SNP at ± 500Kb gene's boundary. **b)** Cross-validation predictive performance upper-bounded to SNP heritability estimate. Red vertical bars indicate mean of the distribution.

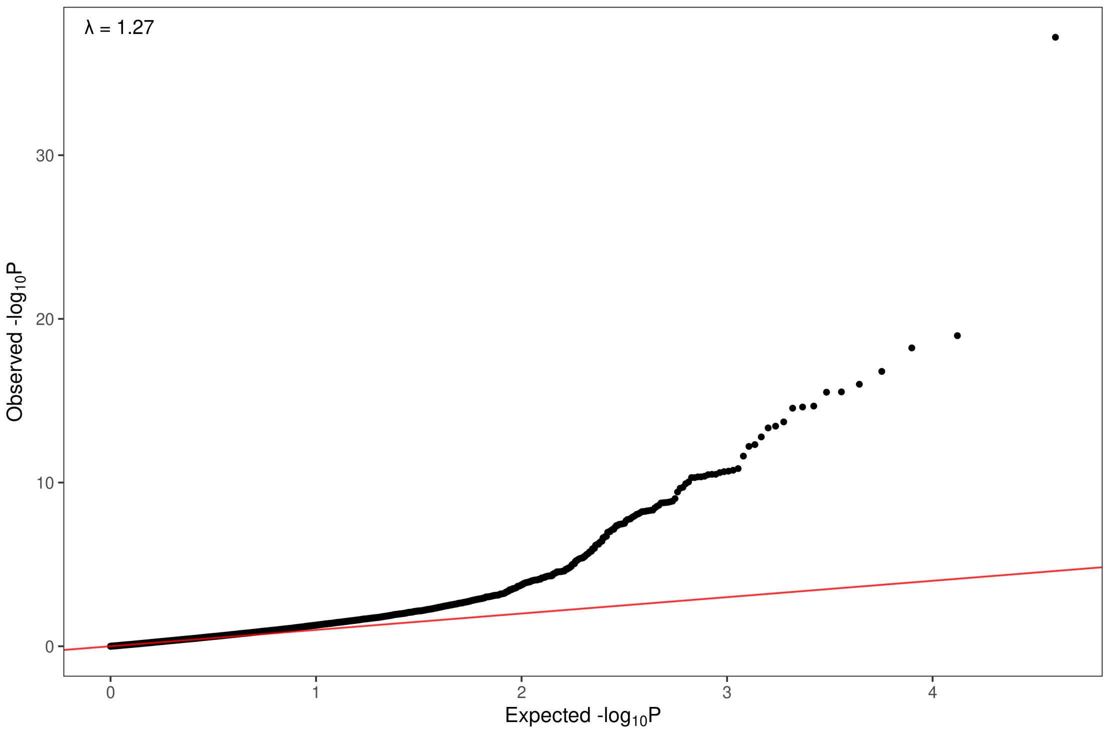

Figure S2. Quantile-quantile P-value plot of the 19,805 TGCT-TWAS association tests.

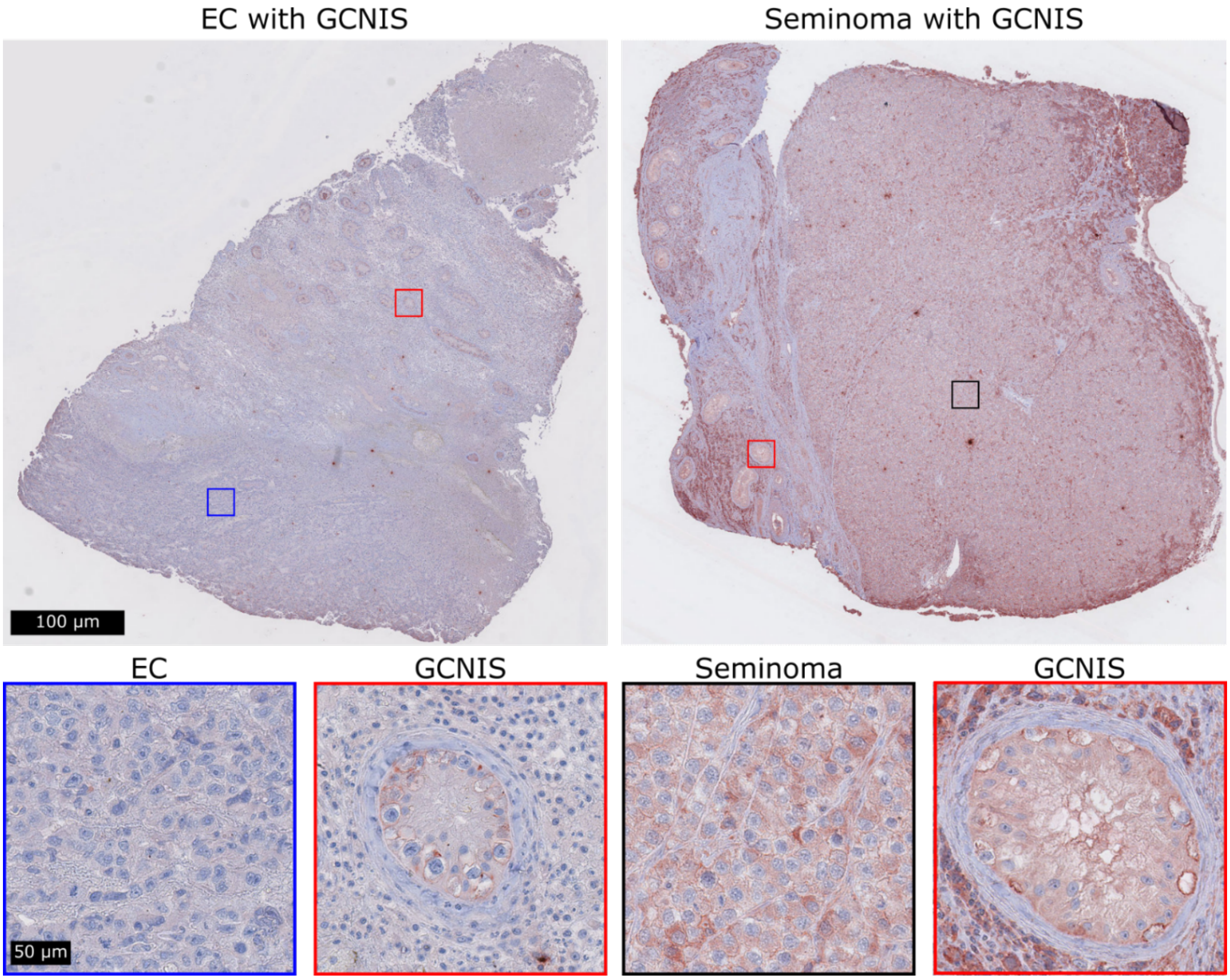

Figure S3. GINM1 protein expression. Protein expression of the novel candidate gene, *GINM1*, in germ cell neoplasia in situ (GCNIS) adjacent to a non-seminoma (embryonal carcinoma, EC) and GCNIS adjacent to a seminoma. On each testicular germ cell tumor subtype, both the tumor and GCNIS component are shown in greater magnification below. *GINM1* was expressed in GCNIS and seminoma, but not expressed in EC. Scale bars indicate 100 and 50 microns, respectively.

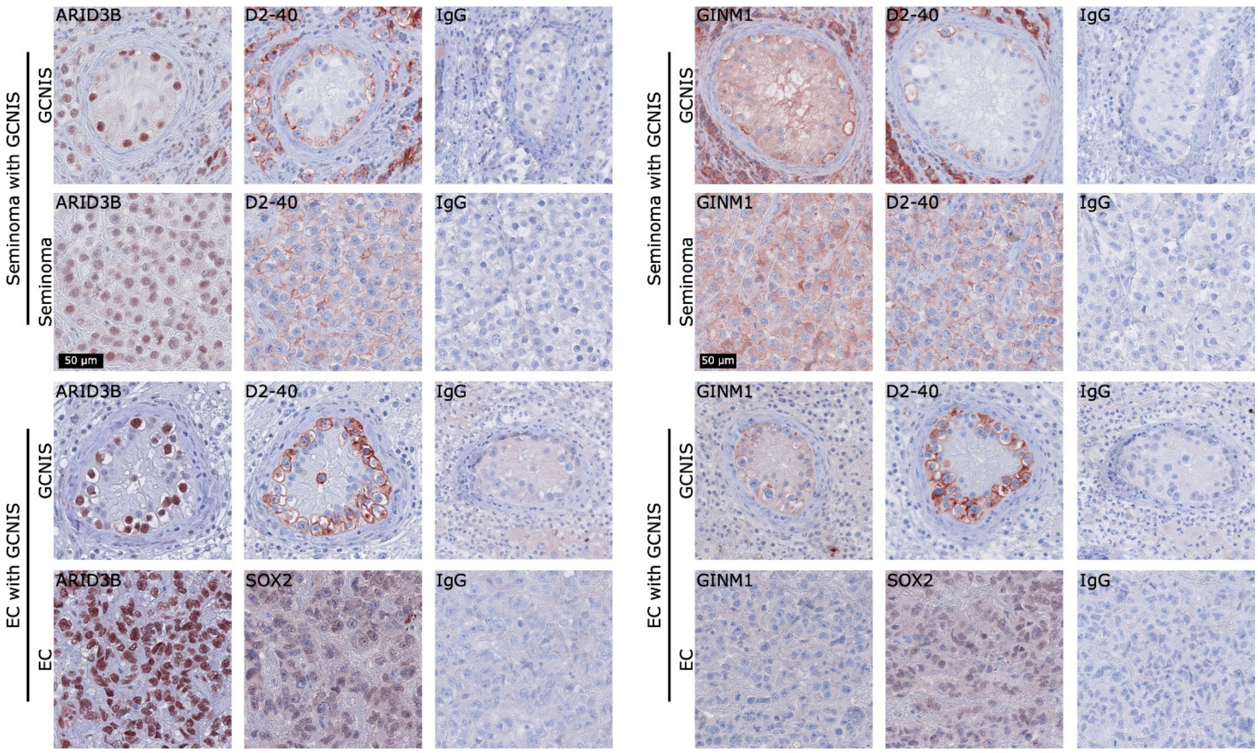

Figure S4. Immunohistochemical markers of tumor components and negative controls. Protein expression of the testicular germ cell tumor markers D2-40 (GCNIS and seminoma), SOX2 (embryonal carcinoma (EC)), and IgG negative control in the magnified areas in Figure 4D (*ARID3B*) and Supplementary Figure 2 (*GINM1*). Note that whereas D240 and SOX2 staining are on serial sections, IgG is not, and the tubules and tumor tissue in the latter therefore looks different than the ones of *ARID3B* and *GINM1* antibodies. Scale bars indicate 50 microns.

Acknowledgments

The Testicular Cancer Consortium is supported by NIH grant U01CA164947 to Katherine L. Nathanson and Peter A. Kanetsky. This work was supported by the Norwegian Cancer Society (208197,418975 and PR-2006-0442), the Nordic Cancer Union (S-12/07), the Swedish Cancer Society (CAN2019/0343) and the Swedish Research Council (2019/011633); Norwegian/Swedish study was supported by the Norwegian Cancer Society (grants number 418975 – 71081 – PR-2006-0387 and PK01-2007-0375), the Nordic Cancer Union (grant number S-12/07) and the Swedish Cancer Society (grant numbers 2008/708, 2010/808, 2011/484, and CAN2012/823). The Penn GWAS (Penn) was supported by the Abramson Cancer Center at the University of Pennsylvania (P30 CA016520), and NIH grant CA114478 to Katherine L. Nathanson and Peter A. Kanetsky; Robert J. Hamilton is supported by the Dell'Elce Family Fund, Princess Margaret Cancer Foundation. The laboratory of Davor Lessel is supported by the Deutsche Krebshilfe grant (70113348). Kevin T. Nead is supported by NIH/NCI MD Anderson Cancer Center Support Grant P30 CA016672. Leeds and Newcastle University's contributions were supported by Cancer Research UK Programme Award C588/A19167. The UK testicular cancer study was supported by the Institute of Cancer Research, Cancer Research UK and made use of control data generated by the Wellcome Trust Case Control Consortium (WTCCC). Stephen M. Schwartz is supported by National Cancer Institute grant R01CA085914 and contracts CN-67009 and PC-35142, and Fred Hutchinson Cancer Research Center institutional funds. Clare Turnbull is supported by the Movember foundation. Tongzhang Zheng receives support from National Cancer Center grant CA104786. A portion of this work was supported by the Intramural Research Program of the National Cancer Institute and by a support services contract HHSN26120130003C with IMS, Inc. Douglas R. Stewart receives support from the Intramural Research Program, National Cancer Institute; Project Number ZO1-CP-10144: Clinical/Genetic Studies of Familial and Hereditary Cancer Syndromes. Anna Gonzalez-Niera receive support from Spanish Ministry of Health Instituto Carlos III-FIS PI17/01822. The EPSAM study was supported by the Piedmont Region, and the Italian Ministry for Education, University and Research under the program "Dipartimenti di Eccellenza 2018–2022" (D15D18000410001). The funding bodies played no direct role in the study. We thank the participants in the study's testicular cancer germ cell studies worldwide who contributed to this study. Alberto Ferlin would like to thank Dr. Maria Santa Rocca for technical assistance. Jourik A. Gietema and Coby Meijer would like to thank Nynke Zwart and Gerrie Steursma for their contributions to the GWAS. Peter A. Kanetsky, David J. Vaughn, and

Kathrine L. Nathanson would like to thank Linda Jacobs and Donna Pucci for their contributions to participant recruitment and the study participants from the University of Pennsylvania. Jeremie Nsengimana would like to thank all study participants and Louise Parkinson for coordinating the recruitment.
